# Short waterlogging events differently affect morphology and photosynthesis of two cucumber (*Cucumis sativus* L.) cultivars

**DOI:** 10.3389/fpls.2022.896244

**Published:** 2022-07-22

**Authors:** Omolayo J. Olorunwa, Bikash Adhikari, Skyler Brazel, Sorina C. Popescu, George V. Popescu, T. Casey Barickman

**Affiliations:** ^1^Department of Plant and Soil Sciences, North Mississippi Research and Extension Center, Mississippi State University, Starkville, MS, United States; ^2^Department of Biochemistry, Molecular Biology, Entomology, and Plant Pathology, Mississippi State University, Starkville, MS, United States; ^3^Institute for Genomics, Biocomputing, and Biotechnology, Mississippi State University, Starkville, MS, United States

**Keywords:** leaf gas exchange, photosynthetic quantum efficiency, infrared gas analysis, hypoxia, chlorophyll fluorescence

## Abstract

Waterlogging induces growth and developmental changes in sensitive crops such as cucumber (*Cucumis sativus* L.) during early plant development. However, information on the physiological mechanisms underpinning the response of cucumber plants to waterlogging conditions is limited. Here, we investigated the effects of 10-day waterlogging stress on the morphology, photosynthesis, and chlorophyll fluorescence parameters in two cultivars of cucumber seedlings. Waterlogging stress hampered cultivars’ growth, biomass accumulation, and photosynthetic capacity. Both cultivars also developed adventitious roots (ARs) after 10 days of waterlogging (DOW). We observed differential responses in the light- and carbon-dependent reactions of photosynthesis, with an increase in light-dependent reactions. At the same time, carbon assimilation was considerably inhibited by waterlogging. Specifically, the CO_2_ assimilation rate (*A*) in leaves was significantly reduced and was caused by a corresponding decrease in stomatal conductance (g_s_). The downregulation of the maximum rate of Rubisco efficiency (V_cmax_) and the maximum rate of photosynthetic electron transport (J_max_) were non-stomatal limiting factors contributing to *A* reduction. Exposure of cucumber to 10 DOW affected the PSII photochemistry by downregulating the PSII quantum yield (Φ_PSII_). The redox state of the primary quinone acceptor in the lake model (1-qL), a measure of the regulatory balance of the light reactions, became more oxidized after 10 DOW, indicating enhanced electron sink capacity despite a reduced *A*. Overall, the results suggest that waterlogging induces alterations in the photochemical apparatus efficiency of cucumber. Thus, developing cultivars that resist inhibition of PSII photochemistry while maintaining carbon metabolism is a potential approach for increasing crops’ tolerance to waterlogged environments.

## Introduction

Plants are obligate aerobes and require molecular oxygen to enable cellular respiration and other essential processes. However, due to waterlogging, complete submergence, and poor soil drainage, plants often suffer from hypoxia and anoxia (Fukao and Bailey-Serres, 2004; Bai et al., 2013). Hypoxic conditions are characterized by anaerobic respiration responses, which facilitate energy deficits of up to 37.5% in plants because oxygen diffusion in waterlogged soil is 10,000 times lower than in well-drained soil (Gibbs and Greenway, 2003). Plants experience a series of physiological changes under hypoxia conditions, such as limited gas exchange (Voesenek and Bailey-Serres, 2015), reduced hydraulic conductivity (Tournaire-Roux et al., 2003), changes in gene expression (Xuewen et al., 2014), and increased oxidative damage (Shabala et al., 2014). Collectively, such changes redirect the energetic resources of the plant and prevent them from reaching its true genetic potential. In addition, anaerobic conditions can adversely affect leaf water potential (schildwacht, 1989), enzymatic activity (Hasanuzzaman et al., 2017), nutrient absorption and assimilation (Arduini et al., 2019), plant growth and development (Pan et al., 2021), and ultimately lead to a decline in crop yields.

Waterlogging affects over 16% of the world’s cultivated land (Ploschuk et al., 2018), and over 17 km^2^ of the land surface is prone to flooding (Voesenek and Sasidharan, 2013). Furthermore, with the rapid climate change, heavy precipitation events are projected to increase by about 7% for every 1°C increase in global warming, leading to increased flood hazard severity (high confidence) (IPCC, 2021). Hence, there is an increasing need to understand the mechanisms of plant tolerance to waterlogging.

Cucumber (*Cucumis sativus* L.) is one of the world’s most important economic vegetable crops. The current estimated global area for cucumber production is approximately 2.3 million hectares, with an annual output of 87.8 million tons and an average yield of 39.3 t/ha (FAO, 2021). However, cucumbers are sensitive to waterlogging stress due to their shallow root system and high oxygen demand (Xuewen et al., 2014; Xu et al., 2016; Qi et al., 2019). The primary root system of cucumber plants quickly deteriorates under waterlogging due to a lack of oxygen, resulting in energy deficits (Gibbs and Greenway, 2003), limited mineral nutrient uptake and acquisition (Arduini et al., 2019), and a corresponding decline in growth and development (Herzog et al., 2016). Previous studies have shown that the emergence of ARs to promote gas exchange and nutrient absorption is a crucial adaptation mechanism utilized by cucumbers under waterlogging (Qi et al., 2019, 2020). In addition, due to rapidly depleted oxygen under waterlogging, root metabolism changes from aerobic respiration to anaerobic fermentation, while the concentration of CO_2_ and ethylene continues to increase (Sairam et al., 2009; Pampana et al., 2016). These conditions usually result in a reduction in ATP production from 36 to 2 moles of glucose metabolized (Sousa and Sodek, 2002; Gibbs and Greenway, 2003). The lack of energy at the root system adversely affects hydraulic conductivity, photosynthetic rate, water, and mineral nutrient uptake, all of which are the result of the disruption of aquaporins function (Tournaire-Roux et al., 2003; Tan et al., 2018). Thus, the regulation of the onset and development of ARs represents a critical tool for the survival and adaptation of plants subjected to waterlogging.

The substantial decline in the rate of photosynthesis is another characteristic feature of plants subjected to waterlogging conditions. Previous studies have demonstrated the sensitivity of photosynthesis in many crops, including cucumber (Barickman et al., 2019), squash (Lin et al., 2020), tomato (Bhatt et al., 2015), and watermelon (Yetisir et al., 2006). For instance, within the first day of waterlogging treatment, cucumber plants’ CO_2_ assimilation rate (*A*) rate declined rapidly (Kang et al., 2009; Barickman et al., 2019). Thus, even in a short period, the significant reduction in *A* under waterlogging conditions could lead to a decline in plant energy reserves, indicating the existence of a shared metabolic pattern. Imperatively, the factors affecting the *A* of plants are primarily divided into stomatal and non-stomatal limitations. Due to limited oxygen under waterlogging conditions, plants close their stomata to maintain plant water status, causing a decline in stomatal conductance (g_s_) and inhibiting the exchange of CO_2_ required by the plant’s basic processes (Voesenek and Bailey-Serres, 2015). Kozlowski (1984) attributed the reduction in g_s_ associated with high CO_2_ concentration during waterlogging to a decrease in root water absorption caused by physical changes in the protoplasm and plasma membrane. Thus, waterlogging impedes the efflux of water, mineral nutrients, and related metabolites (including sucrose, amino acids, proteins, lipids, abscisic acid, etc.) from the root system, resulting in leaf dehydration and stomatal closure. Barickman et al. (2019) reported that a 10-day waterlogging caused a significant reduction in fresh and dry mass (DM) accumulation due to reduce *A*, g_s_, and *E*. Consequently, a reduction in g_s_ eventually leads to a corresponding decrease in *A* and rate of transpiration (*E*) (Pedersen et al., 2013).

Other limitations of photosynthesis in waterlogged conditions are the decline in the activity of ribulose-1,5-bisphosphate carboxylase/oxygenase (Rubisco), chloroplast damage, and loss of leaf pigments (Ahmed et al., 2002; Bansal and Srivastava, 2015). These non-stomatal factors severely inhibit *A* under waterlogging conditions. In addition, waterlogging stress can impair the maximum quantum yield (F_v_/F_m_) of the photosystem (PSII), leading to photoinhibition of the PSII reaction center (Shao et al., 2013; Zhang et al., 2019). The damage of PSII also affects the photosynthetic electron transport chain and changes the amount of light energy directed to organic synthesis, thereby causing damage to the chlorophyll fluorescence parameters (Guidi et al., 2019). Previous studies have noted critical chlorophyll fluorescence parameters, including photochemical quenching (qP) and electron transport rate (ETR), as valuable evaluation tools for understanding cucumbers’ resilience to waterlogging stress.

Currently, the photosynthesis of cucumber under waterlogging has not been thoroughly studied, especially in the seedling stage. Therefore, more information about the growth, development, and physiological responses is worthy of attention, especially the differences in waterlogging tolerance between cultivars and the time-scale response during waterlogging. The present study evaluated the dynamic changes occurring in cucumber seedlings during a 10-day waterlogging period by determining physiological characteristics such as the growth rate, photosynthesis, and chlorophyll fluorescence. The relative effects of waterlogging stress on carbon assimilation and light reactions were assessed as well. The objective of this study was to explore the waterlogging tolerance of cucumber cultivars using morphological and key physiological parameters that affect carbon assimilation, such as gas exchange and chlorophyll fluorescence parameters. We hypothesized that the carbon assimilation rate of cucumber during waterlogging may be affected by stomatal and non-stomatal factors. This hypothesis was tested in two commercial cucumber cultivars exposed to 10 DOW during early developmental growth stages in a controlled environment.

## Materials and methods

### Plant material and growth conditions

The study was conducted from 23 March until 14 May 2021 at Mississippi State University’s Vegetable Physiology Greenhouse and Laboratory Facility on the North Mississippi Research and Extension Center (Verona, MS; 34.1651, –8.7206). Two cucumber cultivars, “Straight 8” and “Marketmore,” were selected for this study. Based on a previous study by Barickman et al. (2019), “Straight 8” exhibited apparent sensitivity to waterlogging stress, while “Marketmore” is a known commercial cultivar with tolerance to environmental stress. Thus, both cultivars were used to determine if they might respond differently to waterlogging tolerance. The selected cultivars have similar growth and life cycle duration of 50–70 days. Seeds of both cucumber cultivars (Wax Seed Company, Amory, MS, United States) were grown in a controlled environmental chamber (Model E-41L2; Percival Scientific, Inc., Perry, IA) under the following conditions: relative humidity of 50%; photoperiod of 16/8 h (light/dark); a temperature of 26/22°C (day/night); and light intensity of average 420 μmol m^–2^ s^–1^. Similar environmental conditions have previously been used in growing cucumber plants under controlled conditions (Barickman et al., 2019; Qi et al., 2019, 2020). Multiple seeds of each cultivar were sown in 10-cm pots and filled with a soil-less potting mixture (Metro Mix 830, F3B, Sun Gro Horticulture, Agawam, MA, United States). An N-P-K (15-3.9-9.9) controlled-release fertilizer (Osmocote; Scotts Miracle-Gro, Marysville, OH, United States) was applied to the substrate at the rate of 5.93 kg.m^–3^. At 7 days after sowing (DAS), seedlings were thinned to one plant per pot.

### Waterlogging treatments

Fourteen days after germination, uniformly emerged seedlings of cucumber at the second vegetative (V2) leaf stage were subjected to two experimental treatments of waterlogging and control (non-waterlogging) treatments for 10 days. Waterlogging treatments were imposed on cucumber plants by placing six pots of each cucumber cultivar into four replicated 11 L containers (Rubbermaid Inc., Wooster, OH, United States). To simulate waterlogging conditions, each container was filled with enough water to reach 3 cm above the substrate surface. Pots containing cucumber plants under control treatments were watered at optimum field capacity as needed. After 10 days of the treatment (DAT), cucumber plants under waterlogging and control treatments were evaluated for morphological and physiological performance.

### Gas exchange measurements

After 10 DAT, photosynthetic and fluorescence parameters were measured on the second most fully expanded trifoliate. The *A*, g_s_, intercellular CO_2_ concentration (C_i_), and *E* were measured *in situ* with chlorophyll fluorescence at the North Mississippi Research and Extension Center (16.00–20:00 CST) using an LI-6800 portable photosynthesis system (LI-COR, Biosciences, Lincoln, NE, United States). Measurements were allowed to match the chamber environment before the values were recorded. The chamber environment was set to match the growth chamber, with 500 μmol m^–2^ s^–1^ of light, 415 ppm of CO_2_ concentration in the air (C_a_), and 50% of relative humidity. Measurements were conducted on six representative cucumber plants of each cultivar subjected to waterlogging and non-waterlogging treatments. The ratio of *A*/g_s_ was used to calculate intrinsic water use efficiency (WUE) (Martin and Ruiz-Torres, 1992). The internal to external CO_2_ ratio was calculated using the *C_*i*_/C_*a*_* relationship, and the stomatal limitation (L_s_) was calculated as 1- *C_*i*_/C_*a*_*.

### Chlorophyll fluorescence measurements

The LI-6800 using pulse-amplitude modulated (PAM) fluorometry with a Multiphase Flash Fluorometer (6800-01A, LI-COR Biosciences, Lincoln, NE, United States) was used to measure the chlorophyll fluorescence at 10 DAT. During predawn hours (7.00–10:00 CST), the minimal fluorescence (F_o_) was measured on the second-most fully expanded leaf using a measuring light (0.005 μmol m^–2^ s^–1^). In dark-adapted leaves, the maximal fluorescence (F_m_) was quantified using a 1-s saturating pulse at 8,000 μmol m^–2^ s^–1^. The leaves were continuously illuminated for 20 min with an actinic light (1,400 μmol m^–2^ s^–1^) to record the steady-state yield of fluorescence (F_s_). Maximal light-adapted fluorescence yield (F_m_′) was determined by 8,000 μmol m^–2^ s^–1^. The actinic light was turned off, and minimal fluorescence yield (F_o_′) in the light-adapted state was determined after 5 s of far-red illumination. The difference between the measured values of F_m_ and F_o_ is the variable fluorescence (F_v_). The chlorophyll fluorescence parameters were calculated using the following formulas (Genty et al., 1989; Maxwell and Johnson, 2000).


$$\frac{{\text{F}}_{\text{v}}}{{\text{F}}_{\text{m}}}=\frac{{\text{F}}_{\text{m}}-{\text{F}}_{\text{o}}}{{\text{F}}_{\text{m}}}$$

ΦPSII=Fm-′FsFm′

$$\Phi_{\text{NPQ}}=\frac{{\text{F}}_{\text{s}}}{{\text{F}}_{\text{m}}{}^{\prime }}-\frac{{\text{F}}_{\text{s}}}{{\text{F}}_{\text{m}}}$$

$$\Phi_{\text{NO}}=\frac{{\text{F}}_{\text{s}}}{{\text{F}}_{\text{m}}}$$

qP=Fm-′FsFm-′Fo′

$$\text{NPQ}=\frac{{\text{F}}_{\text{m}}-{\text{F}}_{\text{m}}{}^{\prime }}{{\text{F}}_{\text{m}}}$$

where F_*v*_/F_m_ is the maximal photochemical efficiency of PSII, Φ_PSII_ is the actual photochemical efficiency of PSII, Φ_NPQ_ is the quantum yield for the energy dissipated *via* Δ pH and xanthophyll-regulated processes, Φ_NO_ is the quantum yield of non-regulated energy dissipated in PSII, and qP and NPQ are photochemical and non-photochemical qP, respectively. ETR was calculated according to Genty et al. (1989).

### Measurement of CO_2_ response

The CO_2_ response curve (*A*/C_i_) measurements were evaluated using the auto program settings in the LI-6800 at 10 DAT. To measure the steady-state response *A*/C_i_, the leaf chamber settings were fixed at 60% relative humidity, the corresponding light intensity of the growth chamber (500 μmol m^–2^ s^–1^), and the temperature was set at 24°C. Using the built-in program on the LI-6800, measurements were taken in a decreasing then ascending bitonic sequence decreasing from ambient (415 ppm) to 50 ppm then to 1,500 ppm, taken measurements at 300, 200, 100, 50, 200, 400, 600, 800, 1,000, 1,200, and 1,500 ppm CO_2_, with early matching, enabled, and wait times 60–90 s between measurements. *A*/C_i_ analyses were performed according to Sharkey et al. (2007), with few changes as portrayed in Olorunwa et al. (2021) using the excel fitting tool 10.0 available at https://landflux.org/tools. The estimated *A*/C_i_ response curve was further utilized to calculate the maximum rate of Rubisco carboxylation efficiency (V_cmax_) and maximum rate of photosynthetic electron transport (J_max_), according to Sharkey et al. (2007). Representative individual curves were fitted separately, and the extracted parameters were averaged across replicates for each treatment.

### Measurement of light curves

For the light response curves of the photosynthetic performance for both cultivars, measurements were taken using the built-in program on the LI-6800 across light intensities of 0, 10, 50, 75, 100, 200, 400, 600, 800, 1,000, 1,200, and 1,500 μmol m^–2^ s^–1^. Minimum and maximum wait times were 120–150 s between measurements with early matching enabled. During the measurements, chamber CO_2_ was held at 415 ppm, temperature at 24 ± 0.5°C, and relative humidity at 60%. The light response curve fitting was done in two parts: first, the optimal equation was determined by plugging the data into the non-rectangular hyperbola-based model of Prioul and Chartier (1977) and identifying the lowest sum of the squares of the errors as described by Lobo et al. (2013). This resulted in the curve by Abe et al. (2009) having the best fit, and various parameters, including light compensation point (I_c_), light saturation point (I_k_), apparent quantum yield (Φ_i_), and the photosynthetic rate at saturated light (P_max_) were calculated from light response curves using solver function of Microsoft Excel furnished by Lobo et al. (2013).

### Morphological performance and plant harvest

Six representative cucumber plants (from each treatment/rep/cultivar) were harvested at the end of the 10-day experiment to obtain morphological performance of the impact of waterlogging stress. Plant’s phenotypic data of leaf number (LN), leaf area (LA), and leaf fresh mass (FM) were evaluated for each treatment combination. LA was measured using the LI-3100 leaf-area meter (Li-Cor Bioscience, Lincoln, NE). Plant component FM was measured from all plants by using a weighing scale. The sample of the plant FM was lyophilized using a FreeZone 2.5 L Freeze Dryer (Labconco Corp., Kansas City, MO, United States) to determine the DM and percent dry mass (%DM).

### Statistical analysis

The experiment was a randomized complete block design with two waterlogging treatments, two cucumber cultivars, four replications, and 12 plants in a factorial arrangement. In total, 192 plants (4 replicates × 2 waterlogging treatments × 2 cucumber cultivars × 12 plants) were utilized in this study. SAS (version 9.4; SAS Institute, Cary, NC) was used to perform the statistical analysis of the data. Replicated values of all morphological and photosynthetic parameters measured in this study were analyzed using a two-way analysis of variance of the generalized linear mixed model (PROC GLIMMIX) to determine the effects of waterlogging treatments, cucumber cultivars, and their interactions. Fisher’s protected least significant difference tests at *p* ≤ 0.05 were employed to test the differences among interactions of cultivars and treatments for measured parameters. The standard errors of the mean were calculated using the pooled error term from the ANOVA table and presented in the figures as error bars. Diagnostic tests, such as Shapiro–Wilk in SAS, were conducted to ensure that treatment variances were statistically equal before pooling. Pearson correlation analysis was utilized to study the relationship between the studied parameters. Graphs were plotted with OriginPro software (version. 9.8.5.204; Origin Lab Corporation, Northampton, MA).

## Results

### Waterlogging induces morphological changes in cucumber cultivars

Supplementary Table 1 shows no significant interaction between cucumber cultivars and waterlogging treatments for all morphological parameters measured except the DM. However, cultivars and waterlogging treatments independently and significantly affected leaf number (LN), LA, and FM.

**FIGURE 1:**
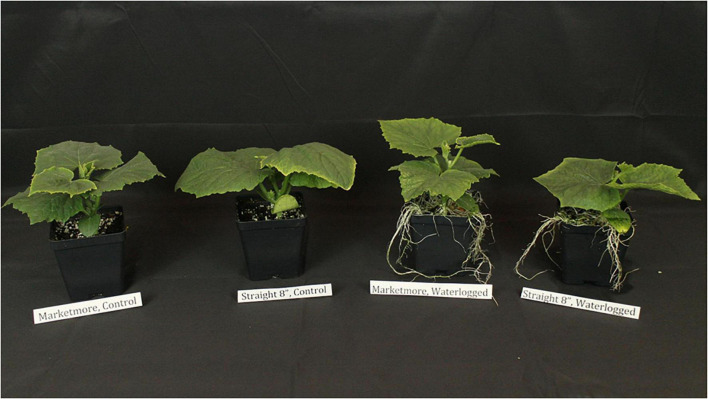
Morphological performance of “Marketmore” and “Straight 8” cucumber cultivars under 10-day control and waterlogging treatments.

**FIGURE 2:**
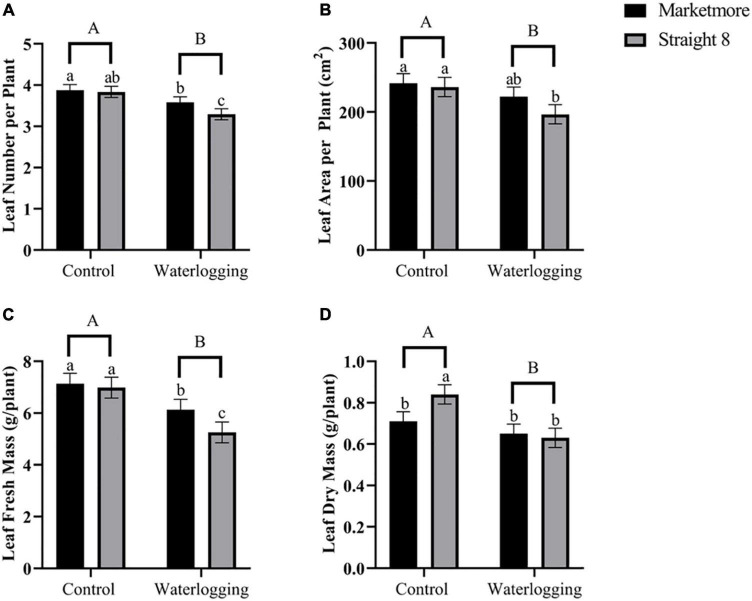
Comparison of **(A)** mean leaf number (LN), **(B)** mean leaf area (LA), **(C)** mean leaf fresh mass (FM), and **(D)** mean leaf dry mass (DM) of cucumber cultivars (Marketmore and Straight 8) after 10 days of control and waterlogging treatments. Different low case and uppercase letters indicate significant differences between the cultivar’s means and treatments, respectively (*P* < 0.05) as determined by Fisher’s LSD. The error bar on the vertical bar indicates the standard error of the mean ± 4 replications of each morphological trait. Standard error of the mean, LN = 0.136; LA = 13.928; FM = 0.403; DM = 0.046.

As displayed in Figure 1, the growth of “Marketmore” and “Straight 8” was affected at 10 DOW, and “Marketmore” was more tolerant than “Straight 8.” The LN, LA, FM, and DM of “Marketmore” decreased by 8, 8, 14, and 8%, respectively, while the same parameters for “Straight 8” were reduced by 14, 17, 25, and 25%, respectively, compared to those of control (Figure 2). The results also revealed that after 10 DOW the cultivars developed ARs primordia on their hypocotyls compared to the control plants (Figure 1).

### Waterlogging induces changes in the photosynthetic performance of cucumber cultivars

**FIGURE 3:**
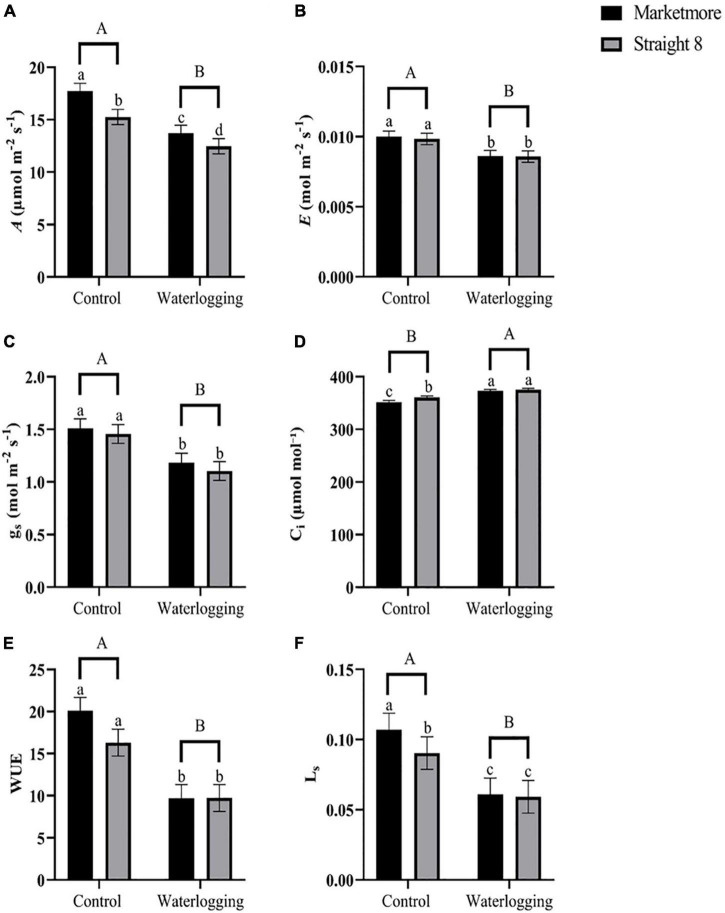
**(A)** CO_2_ assimilation rate (A), **(B)** leaf transpiration rate (E), **(C)** stomatal conductance (g_s_), **(D)** intercellular CO_2_ concentration (C_i_), **(E)** intrinsic water use efficiency (WUE), and **(F)** stomatal limitation (L_s_) of cucumber cultivars (Marketmore and Straight 8) after 10 days of control and waterlogging treatments. Different low case and uppercase letters indicate significant differences between the cultivar’s means and treatments, respectively (*P* < 0.05) as determined by Fisher’s LSD. The error bar on the vertical bar indicates the standard error of the mean ± 4 replications of each leaf gas exchange trait. Standard error of the mean, A = 1.23; E = 0.0007; g_s_ = 0.153; C_i_ = 4.91; WUE = 2.77; L_s_ = 0.0116.

Waterlogging treatments significantly affected all the gas exchange parameters measured between the two selected cultivars, as shown in Supplementary Table 1. The *A* was observed to be significantly lesser in “Straight 8” (12.46 μmol m^–2^ s^–1^) as compared to the “Marketmore,” (13.73 μmol m^–2^ s^–1^) at 10 DOW (Figure 3A). However, g_s_, *E*, and WUE declined to similar levels in the two cultivars: 22, 14, and 52% in “Marketmore,” and 24, 13, and 40% in “Straight 8,” respectively, when compared to the control treatments (Figure 3). In addition, 10 DOW significantly decreased the Ls of “Marketmore” and “Straight 8” by 43 and 34%, respectively (Figure 3F). Interestingly, after 10 DOW, the C_i_ of “Marketmore” and “Straight 8” significantly increased by 6 and 4%, respectively, compared to controls (Figure 3D).

**FIGURE 4:**
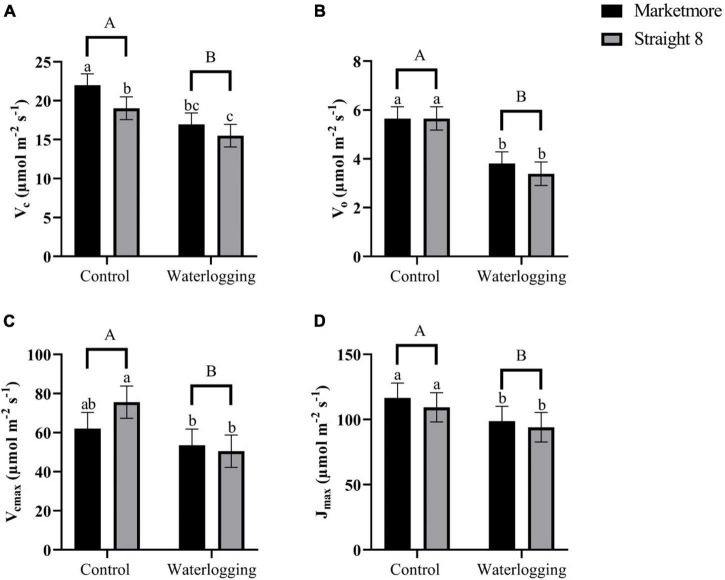
**(A)** Velocity of rubisco carboxylation (V_c_), **(B)** velocity of rubisco oxygenation (V_o_), **(C)** maximum rate of rubisco carboxylation efficiency (V_cmax_), and **(D)** maximum rate of photosynthetic electron transport (J_max_) of cucumber cultivars (Marketmore and Straight 8) after 10 days of control and waterlogging treatments. Different low case and uppercase letters indicate significant differences between the cultivar’s means and treatments, respectively (*P* < 0.05) as determined by Fisher’s LSD. The error bar on the vertical bar indicates the standard error of the mean ± 4 replications of each leaf gas exchange trait. Standard error of the mean, V_c_ = 1.46; V_o_ = 0.478; V_cmax_ = 8.24; J_max_ = 11.30.

**FIGURE 5:**
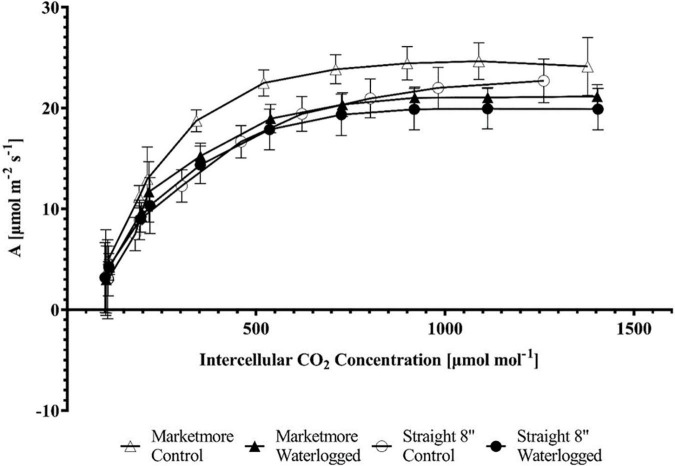
Response of the CO_2_ assimilation rate (A) to increasing intercellular CO_2_ concentration (C_i_) (A/C_i_ Curve) in the two cucumber cultivars (Marketmore and Straight 8) after 10 days of control and waterlogging treatments. The vertical bars represent the standard error of the mean (*n* = 4).

We used the gas exchange data of cucumber cultivars displayed in Figure 3 to estimate the velocity of Rubisco carboxylation (V_c_) and oxygenation (V_o_) (Figures 4A,B) to understand whether the increase in carbon loss from photorespiration could explain the decrease in *A* under waterlogging stress. Both cultivars showed a similar pattern under 10-day waterlogging stress for V_o_ (Figure 4B). However, waterlogging treatment and cultivar independently and significantly affected V_c_ (Supplementary Table 1). Compared to the control treatments, waterlogging significantly decreased V_c_ and V_o_ in “Marketmore” by 23 and 33%, respectively, and by 19 and 28%, respectively, in “Straight 8” (Figures 4A,B). In addition, cultivars and waterlogging treatments significantly and independently influenced the calculated V_cmax_ and J_max_ from the *A*/C_i_ response curve (Figure 5). Under 10 DOW, the V_cmax_ and J_max_ declined by 14 and 15% in “Marketmore” and by 33 and 14% in “Straight 8,” respectively, compared to non-waterlogged plants (Figures 4C,D).

### Waterlogging induces changes in the photochemical efficiency of cucumber cultivars

**FIGURE 6:**
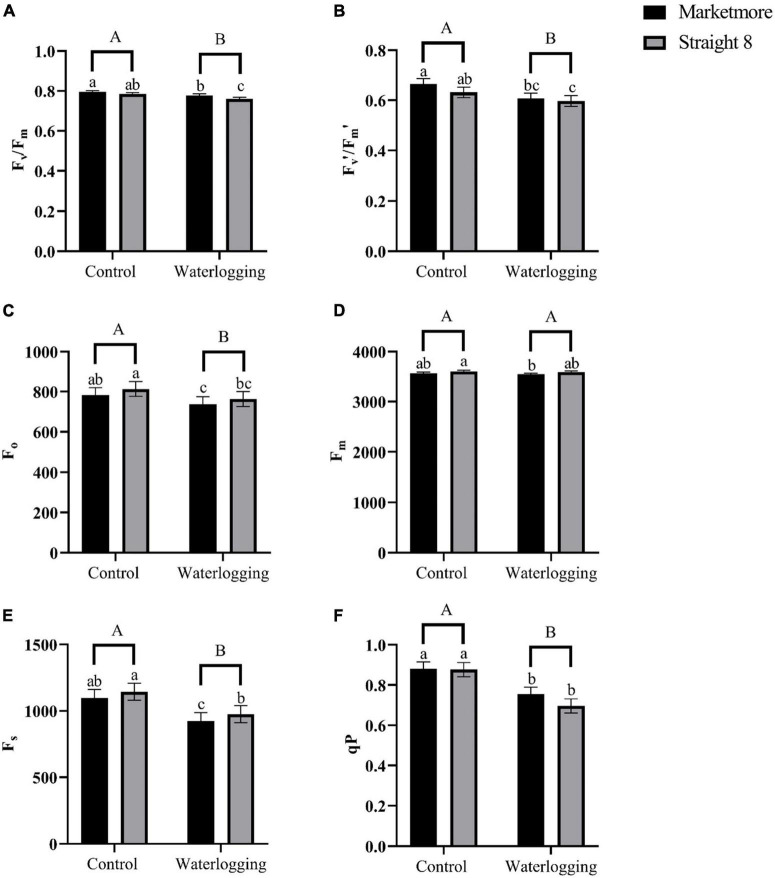
**(A)** Maximum quantum efficiency of PSII in dark-adapted state (F_v_/F_m_), **(B)** maximum quantum efficiency of PSII in the light-adapted state (F_v_′/F_m_′), **(C)** initial fluorescence (F_o_), **(D)** maximum fluorescence (F_m_), **(E)** steady-state fluorescence (F_s_), and **(F)** redox state of the plastoquinone pool (qP) of cucumber cultivars (Marketmore and Straight 8) after 10 days of control and waterlogging treatments. Different low case and uppercase letters indicate significant differences between the cultivar’s means and treatments, respectively (*P* < 0.05) as determined by Fisher’s LSD. The error bar on the vertical bar indicates the standard error of the mean ± 4 replications of each leaf gas exchange trait. Standard error of the mean, F_v_/F_m_ = 0.008; F_v_′/F_m_′ = 0.021; F_o_ = 35.95; F_m_= 85.43; F_s_= 64.09; qP = 0.03.

The values of F_v_/F_m_ following 10 DOW showed the maximum photochemical efficiency slightly declined in “Marketmore” and “Straight 8” compared to the control treatments (Figure 6A). Under waterlogged conditions, the F_v_′/F_m_′ values were found to be comparable for both cultivars (Figure 6B), indicating that PSII was not significantly photoinhibited. In addition, the F_*o*_ values of cucumber cultivars under waterlogging decreased by 5 and 6%, respectively, in “Marketmore” and “Straight 8” (Figure 6C). However, there was no significant difference in the values of F_m_ of both cultivars under waterlogging conditions (Figure 6D). After 10 DOW, the steady state of fluorescence (F_s_) decreased more in “Marketmore” than in “Straight 8” (Figure 6E). The redox state of qP, which measures the fraction of open PSII reaction centers, decreased significantly after 10 DOW treatments in both cultivars, compared to the control (Figure 6F).

**FIGURE 7:**
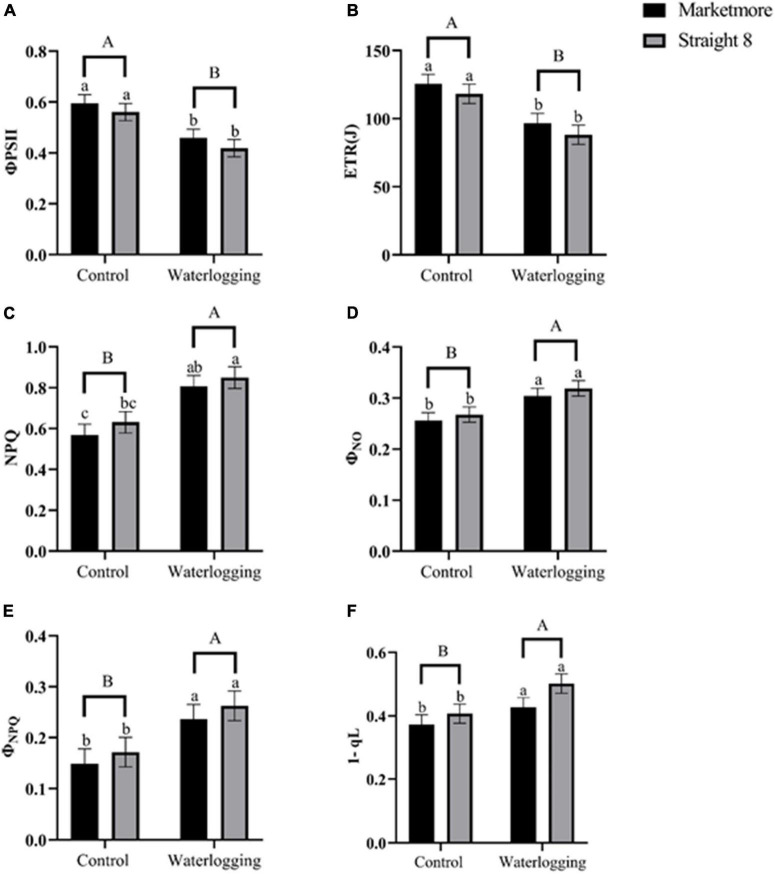
**(A)** Effective quantum yield of PSII (Φ_PSII_), **(B)** electron transport rate (ETR), **(C)** non-photochemical quenching (NPQ), **(D)** quantum yield of non-regulated energy dissipated in PSII (Φ_NO_), **(E)** quantum yield of regulated non-photochemical energy loss in PSII (Φ_NPQ_), and **(F)** redox State of Plastoquinone Pool based on the Lake Model (1-qL) of cucumber cultivars (Marketmore and Straight 8) after 10 days of control and waterlogging treatments. Different low case and uppercase letters indicate significant differences between the cultivar’s means and treatments, respectively (*P* < 0.05) as determined by Fisher’s LSD. The error bar on the vertical bar indicates the standard error of the mean ± 4 replications of each leaf gas exchange trait. Standard error of the mean, Φ_PSII_ = 0.03; ETR = 7.14; NPQ = 0.09; Φ_NO_ = 0.015; Φ_NPQ_= 0.029; 1-qL = 0.03.

Contrarily, alterations in NPQ, which reflect heat dissipation using cucumber leaves, significantly increased after 10 days of waterlogging, compared to the control (Figure 7C). At 10 DOW, the ETR at PSII decreased significantly in “Marketmore” and “Straight 8” by 23 and 26%, respectively, compared to the control treatments (Figure 7B). In addition, we estimated the light energy partitioning at PSII, that is, Φ_PSII_, Φ_NPQ_, and Φ_NO_, which sum to one (Kramer et al., 2004). The Φ_PSII_ of “Marketmore” and “Straight 8” under waterlogging experienced a significant decline of 22 and 25% compared to control treatments (Figure 7A). However, the Φ_NPQ_ and Φ_NO_ increased significantly in both cultivars compared to the control (Figures 7D,E). Furthermore, the redox state of Q_A_ based on the lake model (1-qL) after 10 DOW treatments became more oxidized in both cucumber cultivars compared to the control plants (Figure 7F).

### Waterlogging induces changes in response curves of cucumber cultivars

**FIGURE 8:**
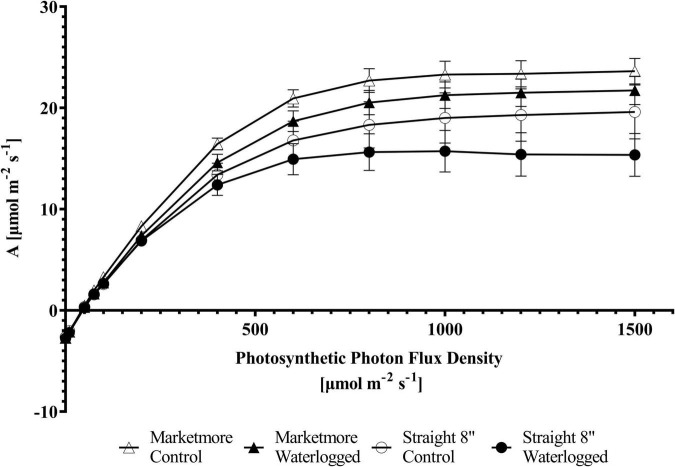
Photosynthetic responses to different light intensities (photosynthetic photon flux density; PPFDs) of the two cucumber cultivars (Marketmore and Straight 8) after 10 days of control and waterlogging treatments. The vertical bars represent the standard error of the mean (*n* = 4).

An *A*/C_i_ curve was constructed to understand the biochemical processes of *A*’s response in cucumbers under waterlogging conditions. The *A* of the cultivars subjected to waterlogging and control treatments increased with increasing C_i_ from 0 to 1,500 μmol mol^–1^ (Figure 5). However, the *A* was lower under waterlogging when compared to the control conditions. A corresponding response was observed with the light response curve. The *A* of cucumbers subjected to 10 DOW or control treatments increased with increasing PPFD from 0 to 1,500 μmol m^–2^ s^–1^ (Figure 8). According to the requirements of PPFD, cucumber plants clearly showed the maximum photosynthetic rate (*A*_max_) under the light intensity of 1,500 μmol m^–2^ s^–1^ under both control and waterlogging treatments. Under control treatment, *A*_max_ was 23.62 ± 1.27 μmol m^–2^ s^–1^ in “Marketmore” and 19.59 ± 2.67 μmol m^–2^ s^–1^ in “Straight 8.” However, *A*_max_ was reduced by 8% under waterlogging in “Marketmore” and reduced by 20% under waterlogging in “Straight 8.”

### Relationships between biomass, gas exchange, and chlorophyll fluorescence parameters

**FIGURE 9:**
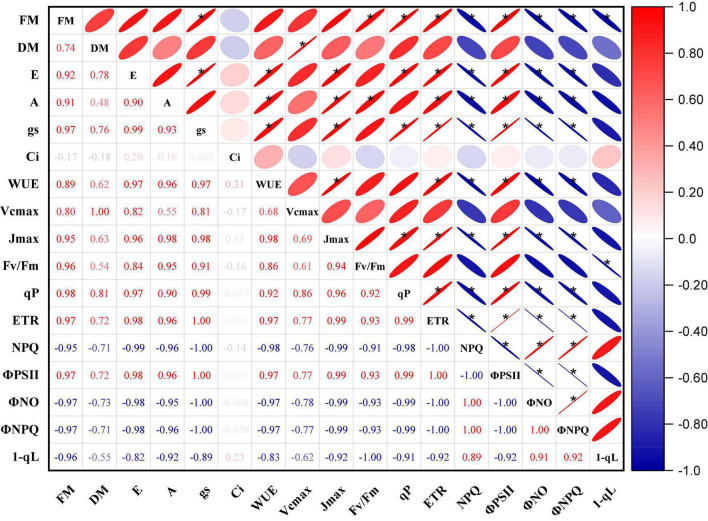
Pearson’s correlation matrix of the changes in biomass, gas exchange, and chlorophyll fluorescence parameters of the two cucumber cultivars under control and waterlogging treatments. Dark color represents strong correlations, and light background color represents weaker correlations. Values close to zero indicate no correlation, and values close to one indicate a strong correlation (positive—red and negative—blue) between two parameters. *Represent correlation coefficient significance levels at *P* ≤ 0.05.

Figure 9 shows the results of a Pearson’s correlation analysis of the biomass, gas exchange, and chlorophyll fluorescence parameters of the “Marketmore” and “Straight 8” under control and waterlogged treatments. The results revealed that the various parameters studied were highly correlated. Except for NPQ, Φ_NO_, Φ_NPQ,_ and 1-qL, which showed a negative correlation, there was a significant and positive correlation between the biomass yields (FM; DM) of cucumbers with the gas exchange parameters. Similarly, most photosynthetic traits (*A*, g_s_, *E*, WUE) under waterlogging treatments were significantly and positively correlated with F_v_/F_m_, qP, ETR, and Φ_PSII_, but negatively associated with NPQ, Φ_NO_, Φ_NPQ,_ and 1-qL. However, the correlation coefficients of C_i_ with most parameters were in the range considered moderate to weak. In addition, V_cmax_ and J_max_ followed the same pattern as the photosynthetic traits, but J_max_ had a stronger positive correlation compared to V_cmax_.

## Discussion

Waterlogging stress imposed on the cucumber plants during the early stages of development results in dramatic changes in plant physiology and biochemistry, with detrimental impacts on morphology and yields. Our previous study on commercially cultivated cucumber plants (“Straight 8”) revealed that the seedling growth stage is extremely sensitive to waterlogging stress (Barickman et al., 2019). We hypothesized that the rapid decline of photosynthetic traits, including *A*, *E*, and g_s_ might contribute to the susceptibility of cucumber plants to waterlogging stress. As an adaptive response to hypoxia, the reduction in the photochemical efficiency of PSII and the formation of ARs could mitigate the potential damage to photosynthetic apparatus caused by waterlogging (Smethurst and Shabala, 2003; Olorunwa et al., 2022). With this hypothesis in mind, we evaluated plant growth, photosynthesis, and chlorophyll fluorescence parameters to identify cultivar-specific and differential responses.

We observed a significant decline in LN, LA, FM, and DM of both cultivars after 10 DOW, demonstrating the sensitivity of cucumber plants at the seedling stage to hypoxia. Previous studies revealed that waterlogging induces morphological changes in plants, especially those related to leaf photosynthesis (Zhang et al., 2019; Olorunwa et al., 2022). Analogous findings have been made in previous studies on cucurbits, including cucumber (Barickman et al., 2019), watermelon (Yetisir et al., 2006), and summer squash (Huang et al., 1995). The alterations in the morphology of cucumber plants under waterlogging can be attributed to reduced water and nutrient uptake and transportation due to energy deficits during anaerobic respiration, resulting in restricted cell expansion and leaf growth (Gibbs and Greenway, 2003; Parent et al., 2008).

The effects of waterlogging stress on leaf morphology and biomass are dependent on plant growth stage, duration, and cultivars. Zheng et al. (2021) reported a significant decline in the waterlogging sensitive “Zaojia8424” leaf biomass compared to the waterlogging tolerant cultivar “YL,” which naturally had smaller leaves. Generally, smaller leaves are more beneficial for mitigating waterlogging stress than larger leaves because of their high boundary layer conductance, which prevents heat accumulation (Leigh et al., 2017). In the current study, 10-day waterlogging reduced the leaf biomass and area in “Straight 8” and “Marketmore” cultivars, with no statistical difference between the two cultivars (Figure 1). In addition, ARs developed in the hypocotyls of both cultivars after 10 DOW. Previous studies showed a similar response for other cucurbits during their early developmental growth stages (Huang et al., 1995; Xu et al., 2016; Qi et al., 2019). The ARs of “Marketmore” was more pronounced after 10 DOW when compared to the “Straight 8” cultivar, mainly due to highly aerenchymatous cortical structures (Visser et al., 1996). In addition, after applying 1-methylcyclopropene (ethylene receptor inhibitor), 1-naphthylphthalamic acid (auxin transport inhibitor), or diphenyleneiodonium (NADPH oxidase inhibitor) to waterlogged cucumbers, Qi et al. (2019) observed the formation of ARs. This implies that auxin, ethylene, and ROS production are enhanced under excess water conditions. Also, Vidoz et al. (2010) suggested that ethylene and auxin might interact in forming ARs in waterlogged tomato hypocotyls using hormone-insensitive mutants. Since auxin and ethylene are transported polarly through ATPase-dependent carrier proteins (Visser et al., 1996), waterlogging-induced low levels of ATP cause these hormones to accumulate near the basal stem of waterlogged plants for solute transport, thereby acting as positive regulators for cucumber waterlogging tolerance.

Waterlogging induces significant changes in the photosynthetic performance of C3 crops, especially in sensitive plants like cucurbits (He et al., 2018; Barickman et al., 2019; Lin et al., 2020). This response was evident in the current study as *A*, g_s_, and *E* for the two cultivars after 10 DOW treatments were significantly reduced. The results of the gas exchange analysis of cucumber plants after 10 DOW indicated that the decline in *A* can be due to both stomatal and non-stomatal factors according to the version of the photosynthesis model by Farquhar et al. (1980). Under waterlogging, the significant decline in *A* and biomass was mainly due to reduced water potential, which resulted in the rapid closure of the stomata through the effect of abscisic acid on potassium ion regulation of guard cell turgor (Sojka, 1992; Ploschuk et al., 2018). Barickman et al. (2019) opined that restricting *A* in cucumber plants through stomatal closure under waterlogging could limit the amount of photoassimilate available for the growth and maintenance of the cucumber vegetative and fruit tissues. In agreement with our results, Issarakraisila et al. (2007) found that the *A* in kale plants was associated with decreased g_s_ after 14 DOW treatments. Although decreased g_s_ is the most common response in plants to waterlogging, the reduction in C_i_ can also be ascribed to non-stomatal factors. The current study showed that g_s_ decreased in two cultivars after 10 DOW, associated with decreased *A* and *E* and increased C_i_. Thus, a significant increase in C_i_ and a decrease in L_s_ in response to changes in *A* and decreased g_s_ under stressed conditions suggest an essential contribution of non-stomatal limitation to *A* (Farquhar and Sharkey, 1982). Previous studies (Yordanova and Popova, 2007; Liu et al., 2014; Pompeiano et al., 2019) in sensitive plants have reported similar findings. In addition, the WUE of both cultivars tested in this study decreased significantly under 10 DOW. Previous research found that waterlogging-sensitive plants have lower WUE and thus gain less carbon per unit of water lost (Meena et al., 2015; Velasco et al., 2019).

In the present study, the reduction in *A* corresponds to the reduction of V_cmax_ and J_max_, which are considered to represent the carboxylation and regeneration of Rubisco, respectively. Previous studies have attributed the decrease in V_cmax_ and J_max_ to the decline in the amount of active Rubisco and a reduction in the contribution of ATP during photosynthesis (Flexas et al., 2014). Urban et al. (2008) considered that changes in *A* response to abiotic stress could be paralleled to the changes in Rubisco activity mediated by J_max_ to balance the modification of V_cmax_ when subjected to stress. In this context, the observed decrease in V_cmax_ and J_max_ for both cultivars after 10 DOW appears to be consistent with a previous study (Ma and Guo, 2014). The *A*/C_i_ further demonstrated that waterlogging stress significantly decreased *A* with increasing C_i_. As per the fitting model of photosynthesis employed in this study (Bernacchi et al., 2001; Sharkey et al., 2007), our results indicated that waterlogging impacted the V_cmax_ more in “Straight 8” when compared to “Marketmore” (33% vs. 14%). Similar results were reported by Ma and Guo (2014) in waterlogging-sensitive cucumber cultivar, and they attributed the significant reduction of V_cmax_ under waterlogging to chlorophyll degradation. Although we did not measure changes in chlorophyll content in the present study, there is increasing evidence that decreases in *A*, V_cmax_, and J_max_ are often associated with the degradation of chlorophyll content in waterlogged plants (Ma and Guo, 2014; Barickman et al., 2019). Primarily, plants subjected to waterlogging stress often experience an increase in the activities of oxidase and chlorophyllase, which are responsible for the degradation of pigments (Muhammad et al., 2021). Jans et al. (2012) suggest that the degradation of plant pigments during waterlogging can be alleviated by reducing the light level capture in a controlled environment. Thus, the downregulation of light utilization efficiency during photosynthesis in a wet environment might be a photoprotective mechanism of cucumber plants.

Moreover, the light response curves revealed that waterlogging declined *A* in cucumber plants, indicating that light utilization was suppressed under waterlogging. Besides the waterlogging stress, for efficient gas exchanges between cucumber leaves and the environment, the optimum PPFD for both cultivars, irrespective of the origin, was 1,500 μmol m^–2^ s^–1^ under the control conditions. However, above the optimum PPFD as depicted in Figure 8, revealed *A* photoinhibition, signifying that an increased PPFD may further hamper cucumber plants’ growth and developmental processes under waterlogging conditions (Wimalasekera, 2019).

Alterations in chlorophyll fluorescence parameters of waterlogged cucumber plants are an adaptive response to waterlogging stress, notwithstanding the photosynthetic apparatus damage. Most of the chlorophyll fluorescence parameters measured in light- and dark-adapted cucumber leaves were significantly reduced under 10-day waterlogging stress, indicating the sensitivity of cucumber plants to hypoxia stress. Previous studies demonstrated waterlogging causes a significant decline in chlorophyll fluorescence parameters, including F_o_, and F_m_, which reflect the PSII electron transport capacity in dark-adapted leaves (Smethurst and Shabala, 2003; Rao et al., 2021; Olorunwa et al., 2022). However, in the current study, although F_o_ decreased, F_m_ did not change significantly after 10 DOW in cucumber plants. The fact that F_m_ did not change significantly in response to waterlogging stress could be attributed to the fact that the degree of water stress was not severe enough to severely damage the leaves or alter metabolic pathways in cucumber plants. Moreover, Krause and Weis (1991) noted that healthy leaves had F_v_/F_m_ values between 0.75 and 0.83, and a drop in these values indicated impaired PSII. However, the results of this study showed that the F_v_/F_m_ of both cucumber genotypes ranged from 0.75 to 0.79 under both control and waterlogging treatments. Our current results are consistent with previous studies (Velasco et al., 2019; Lin et al., 2022), indicating that the alterations in leaf PSII at the seedling stage of cucumber were minimal under waterlogging conditions.

Furthermore, we found that the qP of cucumber plants (representing the proportion of excitation energy dissipated at the expense of photochemical utilization) was significantly reduced under waterlogging stress. Corresponding results have been reported by He et al. (2018) in cucumber plants and Zheng et al. (2021) in watermelon at the seedling stage. These studies attributed the decline in qP to either a slower rate of consumption of reductant and ATP produced by non-cyclic electron transport compared to the rate of excitation of open PSII or damage to PSII reaction centers (Urban et al., 2008). Taken together, the evidence from the current and previous studies further supports the fact that waterlogging leads to a decrease in photorespiration and corresponding damage to the PSII reaction centers of sensitive plants, including cucurbits. Conversely, the NPQ of cucumber plants revealed that the excess excitation energy not used during photochemistry increased in response to waterlogging conditions. Increased NPQ is considered a protective mechanism of the photosynthetic apparatus (Demmig-Adams and Adams, 2018). This result is consistent with previous reports (Ma and Guo, 2014; Bhatt et al., 2015; He et al., 2018) that cucurbit and other fruit crops have increased NPQ and decreased ETR under waterlogged conditions.

According to Kramer et al. (2004), the energy absorbed in PSII is divided into three partitions, including Φ_PSII_, Φ_NPQ_, and Φ_NO_. The current study revealed a decline in Φ_PSII_, and increments in Φ_NPQ_, and Φ_NO_ in cucumber plants under waterlogging, which corroborates with previous studies (Zhang et al., 2019; Zheng et al., 2021). Since the Φ_PSII_ is a product of qP and F_v_′/F_m_′ (Genty et al., 1989), the decrease in Φ_PSII_ under waterlogging may be due to qP and F_v_′/F_m_′ reduction. Moreover, the correlation analysis revealed a positive relationship between Φ_PSII_, qP, and F_v_′/F_m_′. Increases in Φ_NO_ in response to waterlogging indicate the lack of photochemical energy conversion and photoprotective regulatory mechanisms, and the excess electron can be used to form singlet oxygen in the chloroplast (Zheng et al., 2021). In both cultivars, an increase in the 1-qL was also demonstrated in response to waterlogging, suggesting that a more significant proportion of the PSII reaction centers were closed during actinic irradiance, thus resulting in a decline in Φ_PSII_, as denoted by Kramer et al. (2004). The alterations in the qL under waterlogging further indicate that the greater percentage of the energy absorbed in PSII was converted into chemically fixed energy whereas the remaining quanta were dissipated into heat and fluorescence.

## Conclusion

Using morphological and physiological processes, we explored the response of cucumber cultivars to waterlogging stress. The light- and carbon-dependent reactions of photosynthesis were shown to be distinct at the photosynthesis level, with the light-dependent reactions increasing, whereas carbon assimilation was considerably impeded. Decreased stomatal conductance, increased CO_2_ concentration, down-regulated Rubisco carboxylation and regeneration, and absorbed energy dissipated in the form of heat were the main factors that reduced carbon assimilation rate in cucumber plants under waterlogging stress. In addition, the results of this study demonstrated that waterlogging impairs the photoreactions of photosynthesis of cucumber cultivars and that damage could be exacerbated with increasing light intensity.

Overall, our results support the hypothesis that cucumber plants are sensitive to waterlogged conditions, and most effects of waterlogging stress are similar in the two commercial cultivars we analyzed. Notably, the “Marketmore” cultivar appeared more tolerant to waterlogging stress with respect to morphological performance. These observations suggest that cucumber plants can be adapted to waterlogging conditions since they rapidly generate ARs to facilitate gas diffusion and increase plant survival when oxygen concentration is low. However, the newly developed ARs might not contribute to the waterlogging tolerance of cucumbers due to waterlogging-caused inhibition in the carbon metabolism, and PSII reaction centers.

## Data availability statement

The raw data supporting the conclusions of this article will be made available by the authors, without undue reservation.

## Author contributions

OO, BA, SB, and TB designed the experiments. SB collected physiological and morphological data. OO and BA did the statistical analysis using SAS. OO wrote the first draft of the manuscript. SP, GP, and TB made significant comments and inputs to successive versions of the manuscript. All authors contributed to the article and approved the submitted version.

## Conflict of interest

The authors declare that the research was conducted in the absence of any commercial or financial relationships that could be construed as a potential conflict of interest.

## Publisher’s note

All claims expressed in this article are solely those of the authors and do not necessarily represent those of their affiliated organizations, or those of the publisher, the editors and the reviewers. Any product that may be evaluated in this article, or claim that may be made by its manufacturer, is not guaranteed or endorsed by the publisher.
