# Supplementary material for: Short waterlogging events differently affect morphology and photosynthesis of two cucumber (Cucumis sativus L.) cultivars
Source: Front Plant Sci. 2022 Jul 22;13:896244. doi: 10.3389/fpls.2022.896244 (PMC9355484; doi:10.3389/fpls.2022.896244)
Supplement: Supplementary file 1 [file Data_Sheet_1.docx]

**Table S1.** Summary of ANOVA two-way results for morphological and gas exchange parameters of ‘Marketmore’ and ‘Straight 8’ cucumber cultivars under 10 days waterlogging treatments.

| **Studied Parameters** | **Source of variation** | | |
| --- | --- | --- | --- |
|  | Treatment | Cultivar | Treatment * Cultivar |
|  |  |  |  |
| LN (plant ^-1^) | * | *** | NS |
| LA (cm^2^) | * | *** | NS |
| FM (g/plant) | * | *** | NS |
| DM (g/plant) | * | *** | * |
| *A* (µmol m^-2^ s^-1^) | *** | *** | NS |
| g_s_ (mol m^-2^ s^-1^) | *** | NS | NS |
| *E* (mol m^-2^ s^-1^) | *** | NS | NS |
| C_i_ (µmol m^-1^ ) | *** | ** | NS |
| WUE | *** | NS | NS |
| L_s_ | *** | NS | NS |
| ETR | *** | * | NS |
| V_c_ (µmol m^-2^ s^-1^) | *** | * | NS |
| V_o_ (µmol m^-2^ s^-1^) | *** | NS | NS |
| V_cmax_ (µmol m^-2^ s^-1^) | *** | * | NS |
| J_max_ (µmol m^-2^ s^-1^) | *** | * | NS |
| F_v_/F_m_ | *** | * | NS |
| F_m_ | NS | * | NS |
| F_o_ | ** | * | NS |
| F_v_'/F_m_' | *** | ** | NS |
| qP | *** | NS | NS |
| qL | *** | NS | NS |
| 1-qL | *** | NS | NS |
| Φ_PSII_ | *** | NS | NS |
| NPQ | *** | NS | NS |
| Φ_NO_ | *** | NS | NS |
| Φ_NPQ_ | *** | NS | NS |

Note: *, ** and *** represent statistical significance at *P* ≤ .05, .01, and .001, respectively. NS represents not statistically significant *P* > 0.05.

**Table S2.** Water quality of the waterlogged water.

| Parameter | Unit | Values |
| --- | --- | --- |
| pH |  | 6.2 |
| EC | dS·m^−1^ | 1.8 |

EC: Electrical conductivity
